# Supplementary material for: Pressure-induced reconstitution of Fermi surfaces and spin fluctuations in S-substituted FeSe
Source: Sci Rep. 2021 Aug 26;11:17265. doi: 10.1038/s41598-021-96277-9 (PMC8390510; doi:10.1038/s41598-021-96277-9)
Supplement: Supplementary file 1 — Supplementary Information. [file 41598_2021_96277_MOESM1_ESM.pdf]

Supplementary Material – Pressure-induced reconstitution of Fermi surfaces and spin fluctuations in S-substituted FeSe

T. Kuwayama,[1] K. Matsuura,[2] J. Gouchi,[3] Y. Yamakawa,[4] Y. Mizukami,[2] S. Kasahara,[5] Y. Matsuda,[5] T. Shibauchi,[2] H. Kontani,[4] Y. Uwatoko,[3] and N. Fujiwara[1]
[1]*Graduate School of Human and Environmental Studies, Kyoto University, Yoshida-Nihonmatsu-cyo, Sakyo-ku, Kyoto 606-8501, Japan*
[2]*Graduate School of Frontier Sciences, University of Tokyo, 5-1-5 Kashiwanoha, Kashiwa, Chiba 277-8581, Japan*
[3]*Institute for Solid State Physics, University of Tokyo, 5-1-5 Kashiwanoha, Kashiwa, Chiba 277-8581, Japan*
[4]*Department of Physics, Nagoya University, Furo-cho, Nagoya 464-8602, Japan*
[5]*Division of Physics and Astronomy, Graduate School of Science, Kyoto University, Kitashirakawa Oiwake-cho, Sakyo-ku, Kyoto 606-8502, Japan*

Outline

1. **Determination of T_c from the AC susceptibility**
2. **Magnetization**
3. **Orbital part of the Knight shift determined from the K-χ plot**

I. DETERMINATION OF T_c FROM THE AC SUSCEPTIBILITY

We determined T_c from the resonance frequency f_r of the tank circuit attached to the head of an NMR probe. The frequency f_r was measured using a commercially available network analyzer. It is related to the AC susceptibility χ as $f_r = 1/\sqrt{LC(1+4\pi\chi)}$, where C and L represent the capacitance of a variable capacitor and the inductance of a coil wound onto the sample, respectively. Figure 1 shows the T dependence of f_r: f_r increases gradually with decreasing temperature from room temperature, as L of the coil gradually decreases during the cooling process. The drastic increase in f_r occurs at T_c owing to the Meissner effect. We determined T_cs from the crossing points of the dashed lines, as indicated by the red arrows in Fig. 1. T_c at ambient pressure is approximately 9 K. This result is in agreement with T_c determined from magnetization measurements, as described below.

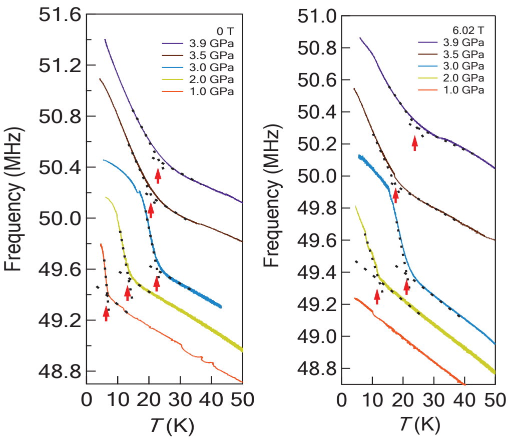

FIG. 1: T dependence of the resonance frequency f_r of the tank circuit measured at zero and 6.02 T. We measured f_r at 6.02 T because the NMR measurements described in the main text were performed at 6.02 T.

II. MAGNETIZATION AND DC SUSCEPTIBILITY

Figure 2 shows the T dependence of the magnetization (M) and susceptibility (χ) measured for 12% S-substituted FeSe with an MPMS system by Quantum Design Inc. T_c determined from the magnetization is in good agreement with that determined from the AC susceptibility. A large demagnetization was observed at $T_c \sim 9K$. The susceptibility data measured at 6 T were used to separate the spin and orbital parts of the Knight shift, as shown in the following K-χ plot.

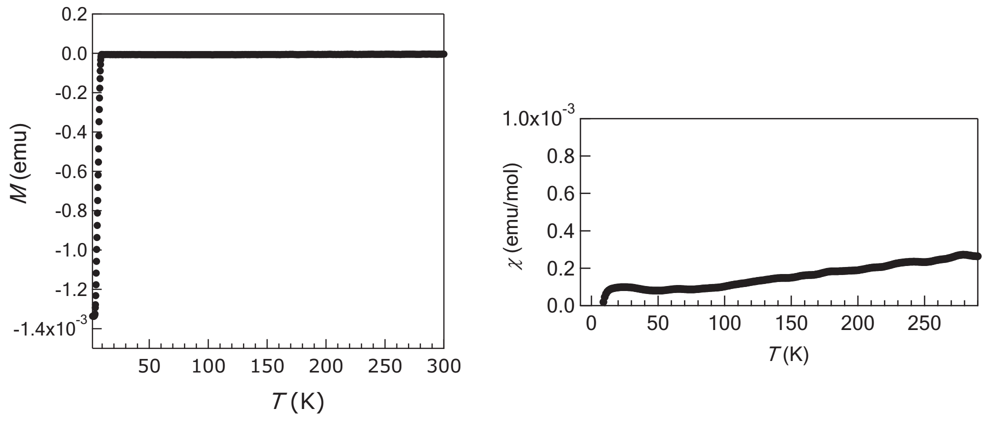

FIG. 2: Magnetization and susceptibility for 12% S-substituted FeSe

III. SPIN AND ORBITAL PARTS OF ^{77}K DETERMINED FROM THE ^{77}K-χ PLOT

The susceptibility χ measured from the experiments is decomposed into three parts: the uniform spin part $\chi(0)$, orbital part $\chi_{\rm orb}$, and diamagnetic term $\chi_{\rm dia}$; $\chi = \chi(0) + \chi_{\rm orb} + \chi_{\rm dia}$. The last term is estimated to be approximately 6×10^{-5} (emu/mol Oe). Both $\chi(0)$ and $\chi_{\rm orb}$ originate from d electrons, and they are estimated only from the ^{57}K-χ plot for ^{57}Fe. $\chi(0)$ and $\chi_{\rm orb}$ are expressed as $^{57}K_{\rm spin} =^{57} A\chi(0)$ and $^{57}K_{\rm orb} =^{57} A_{\rm orb}\chi_{\rm orb}$, where $^{57}K_{\rm spin}$ and $^{57}K_{\rm orb}$ represent the spin and orbital parts of ^{57}K, respectively. ^{57}A is the hyperfine coupling of ^{57}Fe and is obtained from the slope of the ^{57}K-χ plot. $^{57}A_{\rm orb}$ is given as $2 < \frac{1}{r^3} >$, where r represents the distance between an electron on the d orbits and the ^{57}Fe nucleus. Because five d orbitals are involved in iron-based systems, detailed theoretical investigations are needed to restrict the estimation of $^{57}A_{\rm orb}$. Because such theoretical calculations are currently unavailable, $^{57}A_{\rm orb}$ is estimated, for convenience, using $< \frac{1}{r^3} >$ for free Fe^{3+} ions. Five parameters $\chi(0), \chi_{\rm orb}, \chi_{\rm dia},^{57} K_{\rm spin}$, and $^{57}K_{\rm orb}$ are uniquely determined from the following five equations:

$$^{57}K =^{57} K_{\rm orb} +^{57} K_{\rm spin} \quad (1)$$

$$\chi = \chi(0) + \chi_{\rm orb} + \chi_{\rm dia} \quad (2)$$

$$^{57}K_{\rm spin} =^{57} A\chi(0) \quad (3)$$

$$^{57}K_{\rm orb} =^{57} A_{\rm orb}\chi_{\rm orb} \quad (4)$$

$$\chi_{\rm dia} \sim 6 \times 10^{-5} \quad (5)$$

where ^{57}A is determined as the slope of the ^{57}K-χ plot and $^{57}A_{\rm orb}$ is calculated using the parameter of free Fe^{3+} ions. Li *et al.* estimated $\chi_{\rm orb}$ based on the assumption that $\chi_{\rm orb} + \chi_{\rm dia} \sim 0$, namely $\chi \sim \chi(0)$ instead of $^{57}K_{\rm orb} =^{57} A_{\rm orb}\chi_{\rm orb}$ [1].

The Knight shift (^{77}K) of ^{77}Se is obtained from the ^{77}Se-NMR spectra. ^{77}K is decomposed into the orbital part ($^{77}K_{\mathrm{orb}}$) and the spin part ($^{77}K_{\mathrm{spin}}$): $^{77}K =^{77} K_{\mathrm{orb}} +^{77} K_{\mathrm{spin}}$. The latter is related to the uniform spin susceptibility $\chi(0)$; therefore, the density of states ($^{77}K_{\mathrm{spin}} =^{77} A\chi(0)$, where ^{77}A is the hyperfine coupling of ^{77}Se). The ^{77}K-χ plot is often used for the decomposition of ^{77}K into $^{77}K_{\mathrm{orb}}$ and $^{77}K_{\mathrm{spin}}$, as shown in Fig. 3. Li *et al.* also estimated $^{77}K_{\mathrm{orb}}$ to be 0.23% for pure FeSe from the ^{77}K-χ plot under the assumption that $\chi_{\mathrm{orb}} + \chi_{\mathrm{dia}} \sim 0$. The ^{77}K-χ plot for 12% S-substituted FeSe is almost the same as that for pure FeSe [1]; therefore, $^{77}K_{\mathrm{orb}}$ can be estimated to be 0.26%, similar to the case of pure FeSe.

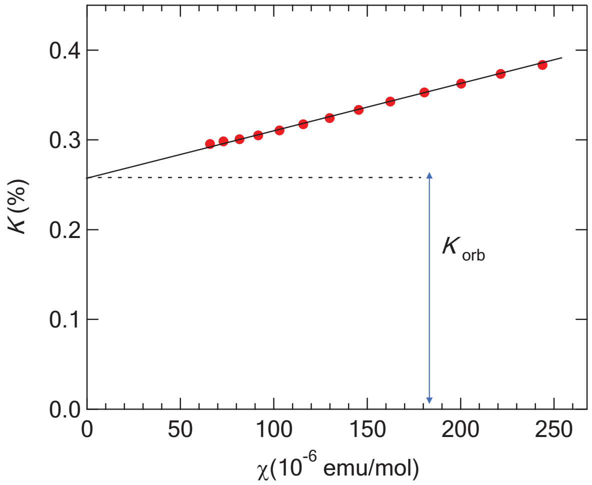

FIG. 3: ^{77}K-χ plot for 12% S-substituted FeSe

[1] J. Li, B. Lei, D. Zhao, L. P. Nie, D. W. Song, L. X. Zheng, S. J. Li, B. L. Kang, X. G. Luo, T. Wu, and X. H. Chen, Supplemental material, Phys. Rev. X 10, 011034 (2020).
